# Report of a Case

**Published:** 1874-08

**Authors:** J. M. Lewis

**Affiliations:** Kosciusko, Mississippi


### REPORT OF A CASE.

BY J. M. LEWIS, M.D., OF MISSISSIPPI.

Kate——, eleven years of age, was salivated when but four (4) years old, and a small portion of her lips had sloughed away. The result of this terrible salivation was anchylosis of the lower jaw, and so completely was her jaws closed that she had to take her food in a liquid form, and through a small opening where a tooth had been extracted (sucking it through). Having to feed herself in this manner, it required most of the day for her to obtain food enough for her system. By moving the inferior maxilla with the hand, it was ascertained that the anchylosis was fibrous or false, but the jaws were held together firmly by adhesive bands on each side of the mouth. These bands, being the result of inflammation, etc., were about one inch wide to about fourteen inches thick. Dr. T. J. Eaton dissected these adhesive bands out, and after completely severing them, he introduced the *beak* of a pair of dental forcips, and with considerable exertion broke up the fibrous adhesions at the joint, and forced the jaws apart. It required considerable force to do this (the bands gave way abruptly, and with some noise.) After opening the jaws, a piece of wood, wedged-shaped, was placed between her teeth to prevent the jaws being closed by the contraction of the tissues healing up. The result was all that could be hoped for. She can open and close her mouth, masticate her food, etc. She could not protrude her tongue more than one-fourth of an inch, but I think she will soon learn the use of that organ well, being a female.

The operation was quite a bloody one, as most operations on the mouth are. Chloroform was used, but with difficulty, the operation being somewhat tedious, and having to suspend the administration of the same, while operating.

Kosciusko, Mississippi.

